# How parental depression influences the development of adolescent depression: based on data from China family panel studies

**DOI:** 10.3389/fpsyg.2025.1514024

**Published:** 2025-04-30

**Authors:** Ruyue Zhai, Shaoqing Yang

**Affiliations:** School of Psychology and Mental Health, North China University of Science and Technology, Tangshan, China

**Keywords:** paternal depression, maternal depression, adolescent depression, development trajectory, latent growth analysis

## Abstract

**Background:**

Previous studies on the impact of paternal and maternal depression on adolescent depression have shown inconsistent findings, and none have examined the influence of parental depression trajectories on adolescent depression trajectories. This study aims to explore the natural developmental patterns of paternal, maternal, and adolescent depression, as well as the predictive effects of parental depression trajectories on adolescent depression trajectories through a longitudinal survey.

**Methods:**

Based on the China Family Panel Studies (CFPS), a total of 1,378 adolescents and their parents were ultimately included in the study. The adolescents and their parents underwent up to three waves of assessments, with each wave separated by 2 years, spanning 6 years (from 2016 to 2020). Latent Growth Modeling (LGM) was used to examine the developmental trajectories of paternal, maternal, and adolescent depression, and the predictive effects of parental depression trajectories on adolescent depression trajectories.

**Results:**

The results of the Latent Growth Curve Analysis revealed an upward trend in the longitudinal measurement of paternal depression (β = 0.483, *p* < 0.001) and adolescent depression (β = 0.318, *p* < 0.001), while maternal depression showed a downward trend (β = −0.340, *p* = 0.015). The results of the parallel process model indicated that the intercept and slope of paternal depression significantly predicted the intercept and slope of adolescent depression, respectively (β_Intercept_ = 0.169, *p* = 0.015; β_Slope_ = 0.488, *p* = 0.008). However, the intercept of paternal depression did not significantly predict the slope of adolescent depression (β = 0.129, *p* > 0.05). Similarly, the intercept and slope of maternal depression significantly predicted the intercept and slope of adolescent depression, respectively (β_Intercept_ = 0.253, *p* < 0.001; β_Slope_ = 0.371, *p* = 0.006). The intercept of maternal depression did not significantly predict the slope of adolescent depression (β = 0.033, *p* > 0.05).

**Conclusion:**

Both paternal and maternal depression should be given equal attention. The developmental trajectories of both paternal and maternal depression influence the developmental trajectory of adolescent depression.

## 1 Introduction

Adolescence is a critical developmental stage. The extensive physiological, social, and emotional transitions make adolescents susceptible to depression (Thapar et al., 2012). Adolescents’ relationships with their parents also change as they strive for independence and autonomy (Laursen and Collins, 2009). Consequently, they are more prone to experiencing exacerbated depressive symptoms (Lewis et al., 2017) and are more vulnerable to the influence of parental depression (Yan et al., 2021). Previous research has indicated that offspring of depressed parents are at least three times more likely to develop depression compared to those with non-depressed parents (Gotlib et al., 2020). Furthermore, adolescents with depression often experience a range of adverse developmental outcomes, such as poorer academic performance and negative peer relationships (Kochel et al., 2012; Verboom et al., 2014). However, previous research has rarely examined the depression of both fathers and mothers simultaneously. Given the negative impacts of depression on adolescents, it is crucial to explore the effects of paternal and maternal depression on adolescent depression.

### 1.1 Predictor: paternal depression, maternal depression

Primary socialization theory (Oetting et al., 1998) emphasizes that the family serves as the primary context for early childhood socialization, with parents acting as the most significant socialization agents. Parental parenting styles, emotional expression, and family environment profoundly influence children’s cognitive, emotional, and behavioral development. A substantial amount of research has explored the relationships between paternal depression, maternal depression, and adolescent depression (Liao et al., 2022; Bi et al., 2024; Chen et al., 2025). However, findings based on different research methodologies have revealed inconsistent associations between adolescent depression and paternal depression (Ohannessian et al., 2005), as well as between adolescent depression and maternal depression (Yang et al., 2017). For example, Hou et al. (2020) and Liao et al. (2022) used longitudinal cross-lagged panel models to explore the relationship between parental depression and adolescent depression. Their research found that maternal depression had a significant predictive effect on adolescent depression, while no direct predictive effect of paternal depression on adolescent depression was observed. However, these two studies have mixed the between-individual and within-individual effects, ignoring between -individual differences, which may overestimate the influence between variables (Mund and Nestler, 2019; Liu, 2021). Furthermore, Bi et al. (2024), based on cross-lagged models, increase the part of the random intercept to isolate between-individual differences, examining the impact of parental depression on adolescent depression within individuals. Their findings revealed that, within individuals, neither paternal nor maternal depression had significant predictive effects on adolescent depression. However, this study has a limitation. Although the model was able to extract both between-individual and within-individual differences, it primarily focused on the within-individual level by controlling for between-individual differences (Hamaker et al., 2015; Murayama et al., 2017). It did not consider exploring the relationships between variables from a between-individual perspective. These traditional methods only compare changes in averages or separate averages into within-individual and between-individual effects, but they fail to provide the complete rate of developmental change for the variables. Latent growth models (LGM) are considered suitable for the between-individual level. It not only extracts intercept factors but also extracts slope factors, allowing for the examination of overall growth trends (Bainter and Howard, 2016; Murayama et al., 2017). Tichovolsky et al. (2018) constructed latent growth models for paternal depression and child depression respectively, estimating the latent intercepts and linear slopes for both paternal and child depression. Parallel process growth curve modeling was used to set predictions in the direction of father depression versus child depression. As a result, the initial level and change rate of paternal depression significantly predicted the change of children’s depression reported by the father. Similarly, Xu et al. (2021) used a parallel developmental model to set the directional prediction of family emotional involvement and older adults’ self-stereotypes, and found that the initial level and rate of change of family emotional involvement predicted the initial level and slope of change of older adults’ stereotypes, respectively. In summary, on the basis of previous research, this study used a parallel development model to set the predictive direction, which will focus on the predicting the development trajectory of paternal and maternal depression on the development of adolescent depression.

In addition, some studies have found that maternal depression trends upward between the ages of 5–21 years for children (Najman et al., 2017). However, to date, most studies of parental depression trajectories have focused on the perinatal period (Larsen et al., 2022; Power et al., 2023), the postpartum year (Volling et al., 2019; Kiviruusu et al., 2020), and parental depression trajectories during childhood (Pietikäinen et al., 2020; Oh et al., 2020). There are relatively few longitudinal studies exploring the natural progression of paternal depression and maternal depression in adolescence. Therefore, this study explores the natural developmental patterns of paternal and maternal depression during adolescence through trajectory analysis.

### 1.2 Paternal depression and adolescent depression

With societal changes and family evolution, fathers are increasingly involved in their children’s upbringing, potentially playing more roles as mentors and playmates within the family. An increasing number of researchers are focusing on the unique role of fathers in children’s psychological development (Reeb and Conger, 2009; Underwood and Waldie, 2017). According to the family system theory, each family member influences others in the same family and is affected by other people (Cox and Paley, 2003). After synthesizing the results of 14 studies, Wickersham et al. (2020) found that the positive association between paternal depression and adolescent depression is as strong as that between maternal depression and adolescent depression. Several longitudinal studies also support the impact of paternal depression on adolescent depression. For instance, Reeb et al. (2010) demonstrated that paternal depression levels can predict adolescent depression levels 1 year later. Lewis et al. (2017) employed a two-sample prospective cohort study, providing evidence for a possible causal relationship between paternal and adolescent depression, showing that a one-standard-deviation (1SD) increase in paternal depressive symptoms is associated with a 0.24 increase in adolescent depressive symptoms. Furthermore, Papp (2012) investigated the trajectories of depressive symptoms among 1,364 adolescents aged 11–15 years in a relatively large community-based family sample and tested whether paternal depressive symptoms moderated these trajectories (i.e., initial levels or slopes). The results indicated that the trajectories of depressive symptoms during adolescence varied with changes in paternal depressive symptoms. In summary, previous studies have consistently shown that paternal depression has an impact on adolescent depression. However, there is a lack of research on the influence of paternal depression trajectories (both intercepts and slopes) on adolescent depression trajectories (including intercepts and slopes). Based on this, this study explores the influence of maternal depression trajectories (including intercepts and slopes) on adolescent depression trajectories (including intercepts and slopes).

### 1.3 Maternal depression and adolescent depression

Based on Family Systems Theory (Cox and Paley, 2003) and the integrated model of intergenerational transmission of maternal depression (Goodman and Gotlib, 1999), mothers serve as the primary caregivers for adolescents in the family. Maternal negative cognitions, emotions, behaviors, and the stressful living environment that they create for their children can all contribute to adolescent depression. Existing research has shown that maternal depressive symptom levels have significant effects on adolescents’ levels of positive affect (PA) and negative affect (NA) (Abitante et al., 2022), depressive symptom levels (Stoolmiller et al., 2005; Brenning et al., 2012), and levels of internalizing problems (Meaney, 2018; Yan et al., 2021).

Several longitudinal studies also support the influence of maternal depression’s developmental changes on the developmental changes of adolescent depression (Kouros and Garber, 2010; Papp, 2012; Pilowsky et al., 2014). Papp (2012) investigated the trajectories of depressive symptoms among 1,364 adolescents aged 11–15 years in a relatively large community-based family sample and tested whether maternal depressive symptoms moderated these trajectories (i.e., initial levels or slopes). The results indicated that the trajectories of depressive symptoms during adolescence varied with changes in maternal depressive symptoms. Pilowsky et al. (2014) conducted baseline depression assessments on 84 mothers with depression and their children. Subsequently, the 84 mothers underwent depression treatment. After a 9-month follow-up, the results found that a decrease in maternal depression significantly predicted a decrease in child depression. Another longitudinal study indicated that for American and Spanish adolescents aged 12–18, decreases or increases in maternal depression levels could significantly predict decreases or increases in adolescent depression (Kouros and Garber, 2010). In summary, previous studies have consistently demonstrated that maternal depression also has an impact on adolescent depression. Similarly, there is a lack of research on the influence of maternal depression trajectories (both intercepts and slopes) on adolescent depression trajectories (including intercepts and slopes). Based on this, this study explores the influence of maternal depression trajectories (including intercepts and slopes) on adolescent depression trajectories (including intercepts and slopes).

### 1.4 The present study

There has been limited research on the stability, developmental changes of paternal and maternal depression, and their relationship with adolescent depression in China. Previous studies have primarily focused on Western countries. Considering the cultural differences between China and the West, it is necessary to explore the impact of parental depression’s developmental changes on adolescent depression’s developmental changes in non-Western countries.

To achieve this goal, the present study employs the Parallel Process Latent Growth Curve Model (PP-LGCM) to explore the dynamic associations between them. The PP-LGCM allows for the simultaneous mapping of covariate trajectories of growth processes for two or more variables and can investigate whether the intercept (i.e., baseline level) and growth (i.e., rate of change over time) of one variable predict the intercept and growth of another variable. In the current study, it permits the simultaneous consideration of the joint development and predictive effects of both parental (father/mother) depression and adolescent depression variables.

This study separately examines the longitudinal prediction of paternal and maternal depression on adolescent depression in China. Based on the limited research results from existing Western samples, this study hypothesizes that paternal and maternal depression levels have a significant positive predictive effect on subsequent adolescent depression (Hypothesis 1). Initially, it investigates the developmental trajectories of paternal depression, maternal depression, and adolescent depression. Finally, it studies the covariate trajectories between paternal and maternal depression and adolescent depression in China.

## 2 Methods

### 2.1 Data

The data utilized in this study were sourced from the China Family Panel Studies (CFPS) conducted by the Institute of Social Science Survey (ISSS) at Peking University. This study recruited families from the 25 most populous provincial regions in China. Due to the absence of depression measurements in 2014, we established 2016 as the baseline and selected 1,375 adolescents aged 10–15 and their parents in that year. This family cohort was then linked with CFPS data from 2018 and 2020. Measurements were conducted every 2 years over a span of 6 years. A total of 1,384 adolescents initially completed the relevant measurements, and after data cleaning, 1,378 participants were ultimately included in the analysis.

During the first survey, a total of 1,375 adolescents participated, with an average age of 11.94 years. Among them, 55.1% were boys and 44.9% were girls. In terms of SES, 9.1% reported belonging to the top 25, 19.9% to the upper-middle 25, 27.4% to the lower-middle 25, and 25.9% to the lowest 25%. Regarding residence, 58.1% were from rural areas, while 40.9% were from urban areas. Additionally, 1,316 fathers were included, with an average age of 40.74 years. In terms of marital status, the majority were married with a spouse (95.7%). Regarding educational attainment, 74.5% had a junior high school education or below, 11.8% had a high school education, and 7.4% had received a university education. Furthermore, 1,333 mothers were included, with an average age of 41.68 years. In terms of marital status, the majority were married with a spouse (96.5%). Regarding educational attainment, 80.1% had a junior high school education or below, 10% had a high school education, and 4.9% had received a university education.

During the second survey, 1,295 adolescents, 1,266 fathers, and 1,299 mothers participated, with attrition rates of 5.81% for adolescents, 3.80% for fathers, and 2.55% for mothers compared to the first survey. During the third survey, 1,046 adolescents, 604 fathers, and 625 mothers participated, with attrition rates of 19.22% for adolescents, 52.29% for fathers, and 51.89% for mothers compared to the second survey. Not all family members completed the individual questionnaires, as some were unreachable, refused to participate, or had other reasons for not completing them.

### 2.2 Measurement

#### 2.2.1 Depression symptoms

Depressive symptoms was assessed using the short version of the Center for Epidemiologic Studies Depression Scale (CES-D) (Radloff, 1977). which included eight items as follows (Zhou et al., 2022): (1) I felt depressed, (2) I felt that everything was an effort, (3) I slept restlessly, (4) I was happy, (5) I felt lonely, (6) I enjoyed life, (7) I felt sad, and (8) I could not get going. Father–mother-adolescent triads self-reported their depression symptoms over the past 7 days on a Likert 4-point scale, ranging from 0 (rarely or none of the time [less than a day]) to 3 (most or all of the time [5–7 days]). The Chinese version of the CES-D has been extensively utilized in previous work (Liu et al., 2023; Jiang et al., 2024). In this study, The Cronbach’s α of adolescent depression scale at three time points were 0.75 ~ 0.78; McDonald’s *ω* is 0.74 ~ 0.78. The Cronbach’s α of paternal depression scale at three time points were 0.78 ~ 0.80; McDonald’s ω is 0.76 ~ 0.79. The Cronbach’s α of maternal depression scale at three time points were 0.80 ~ 0.83. McDonald’s ω is 0.78 ~ 0.81. These results indicate that the internal consistency of the CES-D scale is relatively good across time.

### 2.3 Statistical analyses

The data analysis in this study was conducted using SPSS 26.0 and Mplus 8.3. Latent growth modeling (LGM) was employed to examine the effects of the intercept and slope in paternal and maternal depression on the intercept and slope in adolescent depression, respectively. Firstly, we identified and addressed outliers using the ±3 *σ* rule. Specifically, for each continuous variable, we calculated its mean (*μ*) and standard deviation (σ), and subsequently removed data points falling outside the range of μ ± 3σ. Descriptive statistics, correlation analysis, and reliability analysis for each variable were conducted using SPSS 26.0. Secondly, structural equation modeling was tested using Mplus 8.3. we employed the Full Information Maximum Likelihood (FIML) method to handle missing values, ensuring unbiased and efficient parameter estimation. Unconditional latent growth models for paternal depression, maternal depression, and adolescent depression were constructed to examine their developmental changes. Based on these models, gender and socioeconomic status (SES) were included to build conditional latent growth models for adolescent depression, in order to test the influence of demographic variables on it. Finally, parallel latent growth models for paternal depression, maternal depression, and adolescent depression were constructed to investigate the effects of the levels and changes in paternal and maternal depression on the levels and changes in adolescent depression.

## 3 Results

### 3.1 Descriptive statistics and correlation analysis

There was a significant correlation between paternal depression and adolescent depression (*r*s > 0.09, *p*s < 0.01), and a significant correlation between maternal depression and adolescent depression (*r*s > 0.07, *p*s < 0.05). The specific results are presented in Table 1.

**Table 1:** Descriptive statistics and correlation analysis of variables.

Variables		1. PD			2. MD			3. AD		
		T1	T2	T3	T1	T2	T3	T1	T2	T3
1. PD	T1	1									T2	0.47***	1								T3	0.37***	0.47***	1						
	2. MD	T1	0.30***	0.17***	0.15***	1						T2	0.22***	0.26***	0.20***	0.47***	1					T3	0.17***	0.19***	0.27***	0.43***	0.50***	1			
		3. AD	T1	0.17***	0.05	0.06	0.21***	0.07*	0.12**	1			T2	0.09**	0.14***	0.08	0.08**	0.16***	0.15***	0.25***	1		T3	0.04	0.11***	0.14**	0.06	0.09**	0.12**	0.18***	0.30***	1
Mean				12.51	13.09	13.39	13.33	14.02	13.65	11.76	11.98	12.42
*SD*			3.53	3.53	3.97	3.72	4.05	3.68	2.87	3.01	3.50

PD, Paternal Depression; MD, Maternal Depression; AD, Adolescent Depression; ***p < 0.001, **p < 0.01, *p < 0.05.

### 3.2 Latent growth models for paternal depression, maternal depression, and adolescent depression

Using latent growth curve models, the developmental changes in paternal, maternal, and adolescent depression from T1 to T3 were examined separately. The latent intercept and slope represent the initial level and developmental rate on the trajectory, respectively. The latent intercept factor was modeled based on T1 reports, with the paths from the observed variables at each time point to the intercept factor set to 1. For the latent slope factor, two models were compared: (a) a no-growth model, paths from the observed variable to the slope factor were set to 0; (b) a linear-growth model, paths from the observed variables to the slope factor across the three time points were set to 0, 1, and 2, respectively. Subsequently, conditional growth models were fitted to examine the conditional effects of gender and subjective socioeconomic status (SES) on the intercept and slope of adolescent depression.

#### 3.2.1 Paternal depression

Compared to the no-growth model for paternal depression (*χ*^2^(4) = 52.732, CFI = 0.895, TLI = 0.921, SRMR = 0.052, RMSEA = 0.094, 90%CI = [0.073, 0.118], AIC = 16820.708, BIC = 16846.821), the linear-growth model (*χ*^2^(1) = 2.822, CFI = 0.996, TLI = 0.988, SRMR = 0.014, RMSEA = 0.036, 90% CI = [0.000, 0.090], AIC = 16776.797, BIC = 16818.578) was adopted. The chi-square difference demonstrated a significant improvement in the model fit of the linear-growth model (Δ*χ*^2^ (3) = 49.91, *p* < 0.001). The results indicated that the intercept for paternal depression was 12.552 (*p* < 0.001). The slope for paternal depression was 0.483 (*p* < 0.001), suggesting the linear increase across the three measurement periods. The variance of the intercept (*b* = 6.334, *p* < 0.001) and the variance of the slope (*b* = 0.846, *p* > 0.05) indicated that there were differences in the initial levels of paternal depression among different fathers, but no significant differences in their changes. The correlation between the intercept and the slope was not significant (*r* = −0.506, *p* > 0.05).

#### 3.2.2 Maternal depression

The no-growth model (*χ*^2^(4) = 45.886, CFI = 0.921, TLI = 0.940, SRMR = 0.035, RMSEA = 0.087, 90%CI = [0.066, 0.111], AIC = 17529.703, BIC = 17555.834) and the linear-growth model (*χ*^2^(1) = 23.815, CFI = 0.957, TLI = 0.870, SRMR = 0.037, RMSEA = 0.129, 90% CI = [0.087, 0.176], AIC = 17513.631, BIC = 17555.441) were compared. The chi-square difference indicated that the no-growth model had a relatively better fit (Δ*χ*^2^(3) = 22.071, *p* < 0.001). Considering that both the no-growth model and the linear-growth model exhibited suboptimal fit indices, both models were simultaneously revised. After revision, the fit indices for the no-growth model were as follows: *χ*^2^(3) = 12.733, CFI = 0.982, TLI = 0.982, SRMR = 0.037, RMSEA = 0.049, 90% CI = [0.023, 0.077], AIC = 17498.549, BIC = 17529.907. The revised linear-growth model became a saturated model. The chi-square difference indicated that the fit of the revised linear-growth model was superior to that of the revised no-growth model (Δ*χ*^2^ = 12.733, *p* < 0.01). Therefore, the revised linear-growth model for maternal depression was adopted in this study. The results showed that the intercept for maternal depression was 14.374 (*p* < 0.001), and the slope was −0.340 (*p* = 0.015), indicating the significant decrease in maternal depression across the three measurement periods. The variance of the intercept (*b* = 7.499, *p* < 0.001) and the variance of the slope (*b* = 1.080, *p* = 0.020) were statistically significant, suggesting that there were differences in both the initial levels and changes in maternal depression. The correlation between the intercept and the slope was not significant (*r* = −0.613, *p* > 0.05).

#### 3.2.3 Adolescent depression

Compared to the no-growth model (*χ*^2^ (4) = 44.559, CFI = 0.779, TLI = 0.834, SRMR = 0.060, RMSEA = 0.086, 90% CI = [0.064, 0.109], AIC = 18789.177, BIC = 18815.337), the linear-growth model (*χ*^2^ (1) = 1.494, CFI = 0.997, TLI = 0.992, SRMR = 0.009, RMSEA = 0.019, 90% CI = [0.000, 0.077], AIC = 18752.112, BIC = 18793.968) was maintained. The chi-square difference indicated a significant improvement in the fit of the linear-growth model (Δ*χ*^2^(3) = 43.065, *p* < 0.001). The results indicated that the intercept for adolescent depression was 11.740 (*p* < 0.001), and the rate of change in adolescent depression was 0.318 (*p* < 0.001), suggesting the linear increase across the three measurement periods. The variance of the intercept (*b* = 2.532, *p* < 0.001) and the variance of the slope (*b* = 0.941, *p* = 0.003) indicated that there were differences in both the intercepts and slopes of adolescent depression among different individuals. The correlation between the intercept and the slope was not significant (*r* = −0.370, *p* > 0.05).

**Figure 1:**
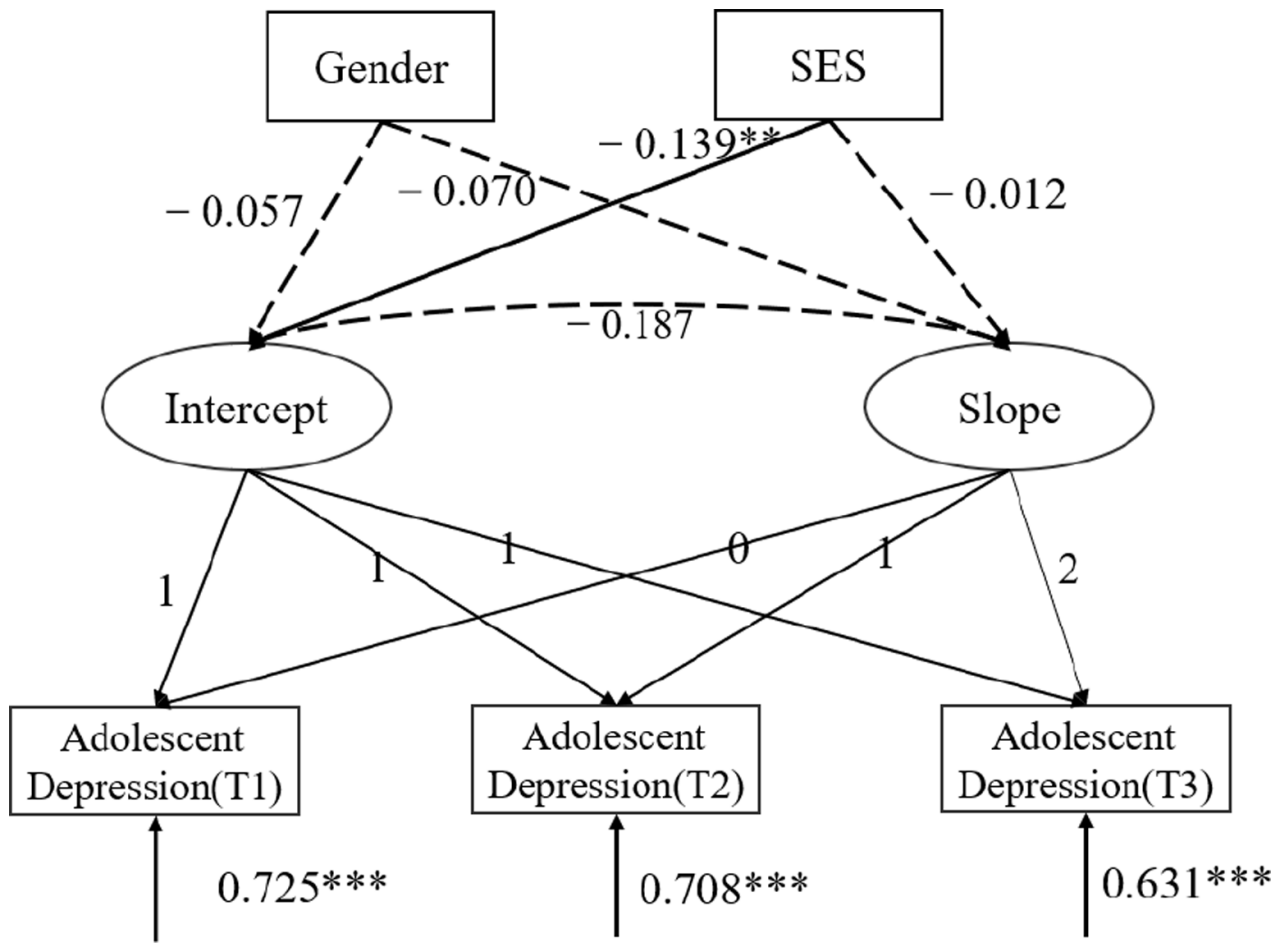
Gender and SES on adolescent depression trajectories. Path coefficients are standardized estimates; Intercept, adolescent depression intercept; Slope, adolescent depression slope; *p < 0.05, **p < 0.01, ***p < 0.001.

Next, we tested the conditional effects of gender and subjective socioeconomic status on the intercept and linear trajectory of adolescent depression. The results showed a model with the following fit indices: *χ*^2^ (3) = 7.829, CFI = 0.971, TLI = 0.913, SRMR = 0.021, RMSEA = 0.036, and 90% CI = [0.001, 0.069]. The results found (as shown in Figure 1) that subjective socioeconomic status had a significant predictive effect on the intercept of adolescent depression (*b* = −0.139, *p* = 0.008), but not on the slope (*b* = −0.012, *p* > 0.05). This indicates that higher subjective socioeconomic status is associated with lower initial levels of adolescent depression. Gender had no significant effect on either the intercept (*b* = −0.057, *p* > 0.05) or the slope (*b* = −0.070, *p* > 0.05) of adolescent depression, suggesting that there are no differences in the intercept and slope between male and female depression.

### 3.3 The parallel process model of adolescent depression and paternal/maternal depression

After establishing the linear trajectories of paternal depression, maternal depression, and adolescent depression, the parallel process model was used to analyze and test the predictive effects of the intercepts and slopes of paternal and maternal depression on the intercepts and slopes of adolescent depression.

**Figure 2:**
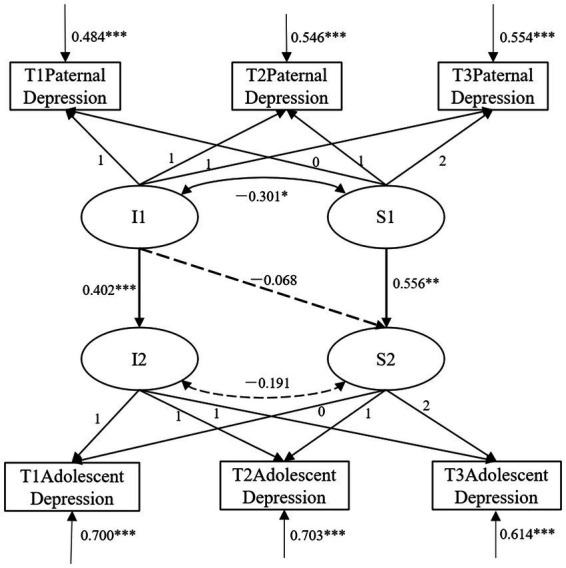
Directional prediction between paternal depression and adolescent depression. Path coefficients are standardized estimates; I1, paternal depression intercept; S1, paternal depression slope; I2, adolescent depression intercept; S2, adolescent depression slope; *p < 0.05, **p < 0.01, ***p < 0.001.

First, in the predictive model of paternal depression on adolescent depression, control for the effects of gender, SES, and maternal depression. The parallel development model showed good fit, with *χ*^2^/df = 2.117, CFI = 0.975, TLI = 0.962, RMSEA = 0.030, 90% CI = [0.015, 0.045], and SRMR = 0.027. The model results (as shown in Figure 2) indicated that the regression coefficient of the intercept of paternal depression on the intercept of adolescent depression was significant (β = 0.402, *SE* = 0.073, *p* < 0.001). This suggests that a higher initial level of paternal depression is associated with a higher initial level of adolescent depression. Additionally, the slope of paternal depression positively predicted the slope of adolescent depression (β = 0.556, *SE* = 0.203, *p* = 0.006), indicating that a faster rate of change in paternal depression is associated with a faster rate of change in adolescent depression over time. However, the initial level of paternal depression did not significantly predict the slope of adolescent depression (β = −0.068, *SE* = 0.117, *p* > 0.05).

**Figure 3:**
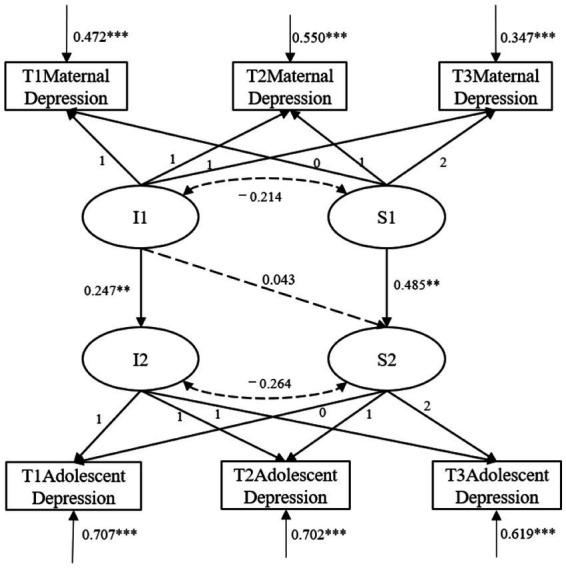
Directional prediction between maternal depression and adolescent depression. Path coefficients are standardized estimates; I1, maternal depression intercept; S1, maternal depression slope; I2, adolescent depression intercept; S2, adolescent depression slope; *p < 0.05, **p < 0.01, ***p < 0.001.

Similarly, in the predictive model of maternal depression on adolescent depression, control for the effects of gender, SES, and paternal depression. The parallel development model showed good fit, with *χ*^2^/df = 2.245, CFI = 0.979, TLI = 0.956, RMSEA = 0.031, 90% CI = [0.016, 0.047], and SRMR = 0.024. The model results (as shown in Figure 3) indicated that the regression coefficient of the intercept of maternal depression on the intercept of adolescent depression was significant (β = 0.247, *SE* = 0.075, *p* = 0.001). This suggests that a higher initial level of maternal depression is associated with a higher initial level of adolescent depression. Furthermore, the slope of maternal depression positively predicted the slope of adolescent depression (β = 0.485, *SE* = 0.165, *p* = 0.003), indicating that a faster rate of change in maternal depression is associated with a faster rate of change in adolescent depression over time. However, the initial level of maternal depression did not significantly predict the slope of adolescent depression (β = 0.043, *SE* = 0.107, *p* > 0.05).

## 4 Discussion

This study investigated the developmental trajectories of paternal, maternal depression and their impact on adolescent depression through a three-wave large-sample survey, thereby strengthening research in this field. It is the first attempt to use a longitudinal study design to explore the influence of both paternal and maternal depression trajectories on adolescent depression trajectories simultaneously. The main findings are as follows.

### 4.1 Developmental patterns of paternal and maternal depression

The LGM results indicated a significant decline in maternal depression over the six-year longitudinal measurement period, which is inconsistent with previous findings (Najman et al., 2017). As children enter adolescence, their needs undergo changes, requiring mothers to continuously adapt to the evolving environment (Hoekzema et al., 2022). The challenges and complexities of the parenting environment may enhance mothers’ cognitive reserve over their lifetime (Orchard et al., 2023), fostering stronger problem-solving skills and emotional regulation abilities (Nomaguchi and Milkie, 2020). Additionally, the specific demands of adolescence may bolster mothers’ resilience (Southwick et al., 2014), promoting psychological growth and role adaptation (Palkovitz, 2019), thereby contributing to the observed decline in maternal depression.

However, paternal depression has shown an increasing trend. Currently, societal expectations of the father’s role have shifted, requiring fathers not only to assume financial responsibilities but also to become more involved in childcare and household duties. On one hand, this multiplicity of roles may elevate the risk of depression (Parent et al., 2019; Rollè et al., 2019). On the other hand, conflicts between work and family, particularly in cases of long working hours or high job stress, may further exacerbate psychological pressure on fathers (Jin et al., 2014; Wang and Peng, 2017). These findings highlight the need for greater attention to the development and changes in paternal depressive symptoms in future research.

### 4.2 Latent changes in adolescent depression

Consistent with previous findings, adolescent depression steadily increased across the three waves of measurement (Bor et al., 2014; Wartberg et al., 2018; Zhou et al., 2024). Prior research has indicated that depression levels tend to rise with age, with older adolescents experiencing a higher prevalence of depressive symptoms compared to younger ones (Wartberg et al., 2018). Despite the scarcity of longitudinal studies on the natural development and changes of adolescent depression in China, a relative consensus can be drawn. That is, at least from primary school to middle school, there is a general upward trend in the level of depression among students.

Previous research has indicated that gender can predict adolescent depression (Liu et al., 2024). However, in the current study, after extracting the intercept and slope of adolescent depression, gender was not found to have a significant predictive effect. In contrast, Yang et al. (2024) found that gender could predict the intercept of adolescent depression, with boys having significantly lower intercepts than girls, which is inconsistent with the findings of the current study. This discrepancy may be influenced by the testing environment. The data of Yang et al. (2024) were collected during the COVID-19 pandemic in 2021–2022. Fear and uncertainty about the disease, the future, and restrictions due to prevention and control measures may have affected adolescents’ emotions, leading to depression. Previous research has indeed found that socioeconomic status (SES) influences adolescent depression (Zeng and Xu, 2024). In the current study, SES negatively predicted the intercept of adolescent depression, indicating that higher SES levels are associated with lower initial levels of depression. However, SES did not significantly predict the slope of adolescent depression, suggesting that it may not have a strong influence on the rate of change in depression over time. Given that there is limited research examining the impact of gender and SES on the trajectory of adolescent depression, there are few results to draw comparisons from. Therefore, future research needs to more comprehensively explore the influence of variables such as gender and SES on the development and changes of adolescent depression.

### 4.3 Paternal depression and adolescent depression

Latent Growth Model (LGM) results provide partial evidence for elucidating the natural coordinated development and changes between paternal depression and adolescent depression. Across six-year assessments, increases in paternal depression levels significantly predicted increases in adolescent depression levels, consistent with previous research findings (Lewis et al., 2017). This result supports the family systems theory. In recent years, an increasing number of fathers have not only taken responsibility for their children’s academic development but have also become more involved in parenting practices (Parent et al., 2019). Therefore, paternal depression is likely to influence the depression levels of their children within the same household.

It is noteworthy that the baseline level of paternal depression does not influence the rate of change in adolescent depression. This suggests that regardless of the initial level of paternal depression, it does not affect how adolescent depression changes over time. Furthermore, the rate of increase in paternal depression significantly affects the rate of increase in adolescent depression, indicating that changes in paternal depression contribute to changes in adolescent depression. This finding aligns with the research results of Papp (2012) but is inconsistent with those of Pilowsky et al. (2014). Regarding the findings of Pilowsky et al. (2014), the results indicated that change in paternal depression do not affect changes in adolescent depression. There may be two possible reasons for this discrepancy. Firstly, Pilowsky et al. (2014) included only 11 fathers in their study, all of whom had severe depressive symptoms. Due to the small sample size and other objective limitations, their study may not have accurately captured the relationship between the two variables. Secondly, cultural differences may play a role. Western peers may emphasize independence and individualism, while China is characterized by a collectivist culture where adolescents often prioritize interdependence and close family relationships (Zhou and Sun, 2021). Paternal depression can affect interactions, relationships, and emotions with adolescents. For example, higher levels of father-son hostility and conflict (Kane and Garber, 2009; Low and Stocker, 2005), as well as more rejecting behaviors and fewer nurturing behaviors (Elgar et al., 2007), can accelerate the development of adolescent depression.

In summary, over time, both the level and the developmental changes in paternal depression positively predict the level and developmental changes in adolescent depression, suggesting to some extent that there may also be intergenerational transmission of depression from fathers to adolescents. Existing research has shown that paternal depression is a potential risk factor associated with an increased risk of depression in offspring, which may be the result of genetic and developmental environmental influences acting alone or in combination (Dachew et al., 2023). Children’s first close observations are often of their parents, so their behaviors and outcomes of their behaviors are the primary objects of their observation. Subsequently, children learn and imitate various behaviors and psychological states of their parents. Therefore, intergenerational transmission occurs subtly in this process.

### 4.4 Maternal depression and adolescent depression

Firstly, the results of the parallel growth model indicate that the intercept of maternal depression positively predicts the intercept of adolescent depression. This is consistent with previous research findings (Monti and Rudolph, 2017). When adolescents are exposed to maternal depression, they exhibit higher initial levels of depression. This result also validates the intergenerational transmission of maternal depression. Secondly, the rate of change in maternal depression influences the rate of change in adolescent depression. This is consistent with previous research findings (Kouros and Garber, 2010; Papp, 2012), suggesting that this result may have cross-cultural universality. Mothers have always been recognized as the primary caregivers and nurturers of their children, with closer emotional communication and bonding with them (Shafer et al., 2017).

Furthermore, the impact of both paternal and maternal depression on adolescent depression also validates primary socialization theory (Oetting et al., 1998). On the one hand, depressed parents may struggle to provide the emotional support and positive guidance that children require due to their own emotional difficulties (Joyner and Beaver, 2020; Murdock et al., 2018; Rinne et al., 2022). On the other hand, adverse family environments and dysfunctional interaction patterns can hinder children’s socioemotional development, leaving them with a lack of security and support (Liu et al., 2020). Consequently, children may develop negative self-perceptions and maladaptive emotion regulation patterns, thereby increasing their risk of depression.

### 4.5 Implication

This study holds significant theoretical implications. First, it reveals the long-term dynamic effects of parental depression on child depression, supporting the perspective of emotional interconnectedness among family members as posited by family systems theory, and further elucidates the long-term trends in this association. Second, this study broadens the perspective of intergenerational transmission theory. We found that the impact of paternal depression on child depression is particularly pronounced during adolescence. While previous research on intergenerational transmission has predominantly focused on maternal depression, this finding highlights that the intergenerational transmission of paternal depression cannot be overlooked. Additionally, it challenges the traditional focus on maternal depression alone, emphasizing the importance of paternal depression and advancing further research on the role of fathers in children’s mental health.

The findings from the current study have important practical implications. First, educators can utilize mental health education to help adolescents develop positive self-perceptions, enhance psychological resilience, and mitigate the negative impact of socioeconomic status on mental health. Second, the results underscore the importance of interventions targeting parental depression, highlighting the critical role of family-based interventions in preventing child depression. Finally, the study contributes to raising public awareness of parental depression and its impact on children’s mental health, reducing stigma, and encouraging families to seek help.

### 4.6 Strengths, limitations and future directions

In summary, this study is the first attempt to use a longitudinal research design to simultaneously explore the natural development trajectories of paternal and maternal depression and their impact on the trajectories of adolescent depression. One advantage of this study is that it documents the concurrent changes in paternal, maternal, and adolescent depression. With both parents increasingly involved in parenting, the mental health issues of both fathers and mothers cannot be ignored. To some extent, paternal depression may also exhibit intergenerational transmission. The results of this study emphasize the importance of including fathers in developmental research. We hope that these findings can guide future research on the role of fathers in the development of adolescent psychopathology. Future preventive and therapeutic interventions for adolescent depression should be family-based, helping depressed fathers and mothers recognize their mental health issues in order to promote the mental well-being of all family members.

There are still some limitations in this study that need to be noted. Firstly, although this study employed a longitudinal research design with three measurement points, the time points were still insufficient to establish a stable causal relationship between paternal and maternal depression and adolescent depression. Furthermore, Although FIML is effective for handling missing data, the relatively high missing rate in T3 parental data suggests that future studies could employ mixed-methods approaches (e.g., qualitative interviews) to supplement missing information. Secondly, the adolescent depression variable was measured using a self-report scale, which reflects participants’ subjective perceptions of their internal experiences. Future studies should incorporate parent/teacher-reported measures to exclude methodological influences that may exaggerate the observed relationships between variables and enhance the robustness of the results. Finally, this study did not assess possible mechanisms underlying the associations between paternal and maternal depression and adolescent depression, such as parental or family conflict. Future research should examine potential mechanisms to gain a deeper understanding of the impact of paternal and maternal depression on adolescent depression.

## 5 Conclusion

Paternal depression and adolescent depression exhibit upward trends, while maternal depression demonstrates a declining trend. Greater attention should be paid to paternal depression. The intercepts and slopes of paternal depression significantly predict the intercepts and slopes of adolescent depression, and similarly, the intercepts and slopes of maternal depression also significantly predict those of adolescent depression. These findings support the family systems theory and the intergenerational transmission model of depression. Regarding preventive and therapeutic interventions for adolescent depression, a family-based approach should be adopted, which simultaneously assists both fathers and mothers in recognizing their psychological issues, thereby promoting the mental health of all family members.

## Data Availability

The original contributions presented in the study are included in the article/supplementary material, further inquiries can be directed to the corresponding author.
